# Oncotype Dx Breast Cancer Assay in Older Patients: A Real Life Cohort

**DOI:** 10.1002/cam4.71386

**Published:** 2025-11-20

**Authors:** Clément Grosnon, Lauren Seknazi, Djamel Ghebriou, Anne Sabaila, David Buob, Mariana Nedelcu, Marjolaine Le Gac, Mathieu Jamelot, Coralie Prebet, Jean‐Pierre Lotz, Joseph Gligorov, Marc‐Antoine Benderra

**Affiliations:** ^1^ Medical Oncology Department Institut Universitaire de Cancérologie AP‐HP Sorbonne Université, Tenon Hospital Paris France; ^2^ Gynaecology Department Institut Universitaire de Cancérologie AP‐HP Sorbonne Université, Tenon Hospital Paris France; ^3^ Anatomopathology Department Institut Universitaire de Cancérologie AP‐HP Sorbonne Université, Tenon Hospital Paris France

**Keywords:** adjuvant chemotherapy, breast cancer, older patients, Oncotype DX

## Abstract

**Introduction:**

The Oncotype DX Breast Recurrence Score test was designed for HR+, HER2− early breast cancer (eBC) to assist in the decision‐making process for de‐escalating adjuvant chemotherapy (CT). Its validity and utility have been demonstrated prospectively across multiple studies, though data on older patients remain limited.

**Methods:**

This prospective cohort study included patients over 70 years with eBC treated between 2018 and 2023 at Tenon Hospital, Paris, France. Characteristics of populations eligible for the test and those with RS ≤ or > 25 were collected, along with oncogeriatric assessment and treatments; statistical analysis was performed for IDFS and OS.

**Results:**

Of the 365 patients > 70 years, mean age was 77.6 years (range 70–96), with 84.4% diagnosed with HR+/HER2− eBC. Oncotype DX testing was performed in 85 patients (27.9% of HR+/HER2− eBC), revealing a Recurrence Score (RS) result > 25 in 14 patients (15%). The RS > 25 group had more grade 3 tumors, more pT2 tumors, and a higher Ki67 > 20%. In the RS > 25 group, 9 patients (64%) had oncogeriatric consultations, and 7 (50%) started the recommended adjuvant CT. Four patients received taxane‐cyclophosphamide (TC) and 3 adriamycin‐cyclophosphamide‐taxane (AC‐T), with 2 discontinuing due to taxane‐induced neuropathy. Median IDFS was significantly lower in the RS > 25 group (*p* = 0.0023), with 4 of 5 recurrences occurring in patients who did not receive adjuvant CT. OS showed no significant difference by RS group (*p* = 0.87).

**Conclusion:**

This observational study highlights that the Oncotype DX test supports therapeutic de‐escalation in patients with RS ≤ 25 and serves as a prognostic marker. Oncogeriatric evaluations are essential to guide adjuvant treatments in older breast cancer patients prior to using genomic signatures.

## 1 Introduction

According to the World Health Organization (WHO) and Global Cancer Statistics (GLOBOCAN), over two million new breast cancer (BC) cases are reported globally each year, making it one of the most prevalent cancers worldwide [1]. BC classification is based on the expression of estrogen and progesterone receptors (ER and PR) and the human epidermal growth factor receptor 2 (HER2) [2]. For hormone receptor‐positive (HR+), HER2‐negative tumors, adjuvant treatment is guided by clinical and biological factors such as age, lymph node status, tumor size, histological type, grade, and proliferation. Endocrine therapy (ET) is standard except for very small tumors, while chemotherapy (CT) and targeted therapy are considered based on recurrence risk [3, 4].

When combining CT with ET, identifying patients who will benefit from CT is critical. To address this, the 21‐gene Oncotype DX Breast Recurrence Score test was developed to personalize and de‐escalate adjuvant CT decisions for early‐stage HR+ HER2− BC patients [5]. This test calculates a Recurrence Score (RS) result from 0 to 100, with higher scores indicating a greater risk of recurrence. In postmenopausal women with HR+ HER2− early breast cancer (eBC), CT is recommended if the RS result exceeds 25 based on multiple studies.

The TAILORx study demonstrated that for HR+ HER2− N0 eBC, adding CT to ET provides no additional benefit in most cases, sparing patients from unnecessary treatment, with only an RS > 25 indicating a benefit from CT in postmenopausal women [6]. Complementing this, the RxPONDER study found that postmenopausal women with HR+ HER2− eBC and 1–3 positive lymph nodes with an RS < 25 can safely avoid CT regardless of node number, tumor grade, or size [7]. Decision‐impact studies have further demonstrated the clinical and economic benefits of the Oncotype DX test in routine care [8, 9, 10, 11]. Supported by level 1a evidence, it is included in guidelines such as ESMO [3], NCCN [4], and ASCO [12].

Although nearly 30% of BC cases occur in women over 70 [13], data for this group remain limited. In TAILORx, only 7% of participants were over 70, and in RxPONDER, 17%. Similarly, in the PONDx study—a French multicenter investigation of Oncotype DX test's impact—14% of participants were over 70, limiting interpretation for this subgroup [14]. Consequently, the French National Authority for Health (HAS) restricts Oncotype DX use to patients under 70. Due to limited evidence for patients over 70 and the marginal benefit of CT in this group, the Oncotype DX test will no longer be reimbursed for them in France after 2024.

This cohort study aimed to evaluate the use of the Oncotype DX test in patients over 70 and assess distant recurrence and BC‐specific mortality (BCSM) rates by RS category.

## 2 Methods

### 2.1 Design and Ethical Approval

This monocentric prospective cohort study included women over 70 years diagnosed with eBC who underwent breast surgery at Tenon Hospital, Paris, between January 2018 and December 2023. The study received ethical approval from the AP‐HP Data Protection Office (DPO) and was registered in the AP‐HP General Register of Data Processing on January 30, 2025 (registration number: N°20250130150713), in compliance with the General Data Protection Regulation (GDPR) requirements; it adhered to the conformity checklist for research not involving human participants. Informed consent was obtained from all participants included in the study.

### 2.2 Data Collection

Patient and tumor data were extracted from medical records, including Tumor Board Meeting and pathology reports, as well as oncogeriatric assessments. Collected data encompassed surgical procedure type, *BRCA1/2* mutation status (if tested), histological classification (ductal or lobular), Elston‐Ellis tumor grade, postoperative TN stage (pTN), and presence of vascular or lymphatic emboli. HR status was considered positive based on standardized European guidelines with a 10% cutoff of stained tumor nuclei. HER2 status was evaluated using IHC and FISH for HER2 2+ scores, with HER2 positivity defined as a 3+ score or 2+ with positive FISH.

Oncotype DX testing was performed after validation during a multidisciplinary team (MDT) meeting, in accordance with international guidelines [12]. It was not performed in patients who were not eligible for chemotherapy due to significant frailty.

RS results and treatment recommendations—such as radiotherapy, ET, CT, or other modalities—were documented following test availability. An RS > 25 prompted a recommendation for adjuvant chemotherapy in both N0 and N1 patients, pending oncogeriatric evaluation and patient consent. Although this cut‐off has not been prospectively validated in patients aged over 70 years, it corresponds to the threshold established by the TAILORx and RxPONDER trials [6, 7] and is therefore widely adopted in geriatric oncology practice [15]. Oncodage G8 scores and details of oncogeriatric assessments were recorded. For patients with RS > 25, data on CT initiation or refusal were collected, including reasons for non‐initiation. The type of CT administered and premature treatment discontinuation were also documented.

### 2.3 Objectives and Outcomes

Primary objectives included the description of the patient population in which Oncotype DX was performed and the analysis of the subgroup of patients with RS ≤ 25 and > 25 including the geriatric assessment. Secondary objectives included statistical analysis on invasive disease‐free survival (IDFS) and overall survival (OS) according to RS. IDFS was defined as the time from the date of surgery to the occurrence of any invasive cancer recurrence (local, regional, or distant) or death from any cause. OS was defined as the time from the date of surgery to death from any cause. The date of recurrence, date of death, and cause of death were recorded when known. BC‐related deaths were defined as those occurring in patients with metastatic disease at the time of death. The median follow‐up duration was calculated from the date of surgery to the censoring date, defined as either the date of death or the last follow‐up, with data collection concluding on October 20, 2024.

### 2.4 Statistical Analysis

Statistical analyses were performed for the full population and for the subpopulation that underwent Oncotype DX testing. Survival analyses using the Kaplan–Meier method, including IDFS and OS, were performed on the RS ≤ 25 and RS > 25 groups. Continuous variables were reported as mean ± standard deviation (SD) for normally distributed data or as median [interquartile range (IQR)] or median (range) for non‐normally distributed data. Categorical variables were expressed as frequency (percentage). The log‐rank test was employed to compare IDFS and OS between RS groups. Hazard ratios (HR) and 95% confidence intervals (CI) were computed using Cox regression models to evaluate the association between RS groups (≤ 25 vs. > 25). Patients were censored at the time of the last follow‐up, the date of medical records review, or the time of death (from any cause). The threshold for statistical significance was set at *p* < 0.05 and no formal statistical power calculations were performed. All tests were two‐tailed, and statistical analyses were conducted using R software (version 4.0.3).

## 3 Results

**FIGURE 1:**
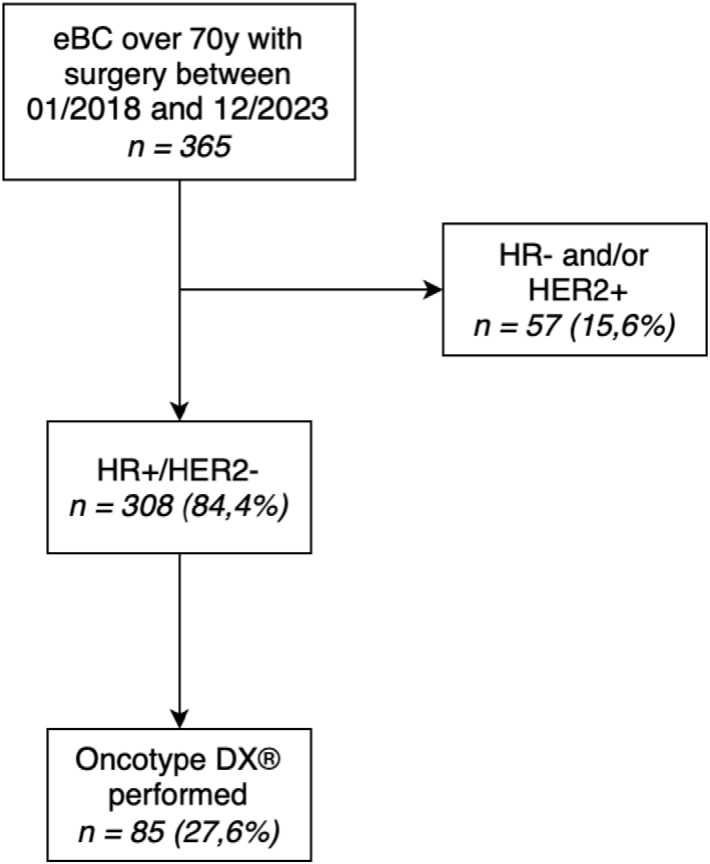
Study flowchart. EBC, early breast cancer; HR, hormone receptor; RS, recurrence score; y, years.

A total of 365 patients over 70 years were included in this study. Among them, 308 patients (84.4%) were HR+/HER2−, representing the primary population of interest for the Oncotype DX test, and 85 patients (27.6%) underwent the Oncotype DX test (Figure 1).

Characteristics of the population included in the study are provided in Table 1. The mean age was 76 years (range 70–96) for the entire cohort; all patients were female (100%). Histological characteristics were globally similar across the three groups, with a predominance of invasive ductal carcinoma (IDC), although invasive lobular carcinoma (ILC) was more frequent in the Oncotype DX group. BRCA1/2 mutations were rare across groups.

**TABLE 1:** Patient and disease characteristics of the total population, the HR+/HER2− population and the Oncotype DX test performed population.

	Total population *n* = 365	HR+/HER2− population *n* = 308	Oncotype DX performed *n* = 85
Median of age (IQR)	76 (9)	76 (9)	74 (6.5)
IDC	274 (75%)	226 (73.4%)	58 (68.2%)
ILC	91 (25%)	82 (26.6%)	27 (31.8%)
*gmBRCA1/2* ^a^	3 (1%)	3 (1%)	0 (0%)
Mastectomy	154 (42%)	123 (39.9%)	33 (38.8%)
Tumorectomy	211 (58%)	185 (60.1%)	52 (61.2%)
Adjuvant radiotherapy	278 (76.2%)	234 (76%)	72 (84.7%)
Adjuvant hormonotherapy	315 (86.3%)	295 (95.8%)	84 (98.8%)
pT0	6 (1.6%)	2 (0.6%)	0 (0%)
pT1	234 (64.1%)	196 (63.6%)	39 (45.9%)
pT2	105 (28.8%)	92 (29.9%)	42 (49.4%)
pT3	13 (3.6%)	12 (3.9%)	4 (4.7%)
pT4	7 (1.9%)	6 (2%)	0 (0%)
pN0	278 (76.2%)	237 (76.9%)	50 (58.8%)
pN1	72 (19.7%)	57 (18.5%)	35 (41.2%)
pN2	9 (2.5%)	8 (2.6%)	0 (0%)
pN3	6 (1.6%)	6 (1.9%)	0 (0%)
Grade 1	76 (20.8%)	72 (23.4%)	8 (9.4%)
Grade 2	229 (62.7%)	204 (66.2%)	62 (72.9%)
Grade 3	60 (16.4%)	32 (10.4%)	15 (17.7%)
HR+/HER2neg	308 (84%)	308 (100%)	85 (100%)
HER2pos	15 (4%)	0 (0%)	0 (0%)
Ki67 > 20%	92 (25.2%)	55 (17.9%)	31 (36.5%)
Vascular and/or lymphatic invasion	49 (13.4%)	40 (13%)	22 (25.9%)

Abbreviations: BC, breast cancer; gm, germline mutation; HR+, hormone receptor positive; IDC, invasive ductal carcinoma; IHC, immunohistochemistry; ILC, invasive lobular carcinoma; IQR, inter‐quartile range; pN, histopathological node stage; pT, histopathological tumor stage.

^a^ The BRCA1/2 mutation had not been tested for most of the patients.

Focusing on HR+/HER2− and Oncotype DX: grade 2 (72.9%) and grade 3 (17.7%) tumors predominated in the Oncotype DX group, while grade 1 was more frequent in HR+/HER2− (23.4%). T2 tumors were more common in the Oncotype DX group (49.4%), whereas T1 predominated in HR+/HER2− (63.6%). N0 cases were fewer in the Oncotype DX group (58.8%) compared to HR+/HER2− (76.9%), with more N1 cases in Oncotype DX (41.2%). Surgery type, Ki67, and emboli presence showed minor intergroup differences.

**FIGURE 2:**
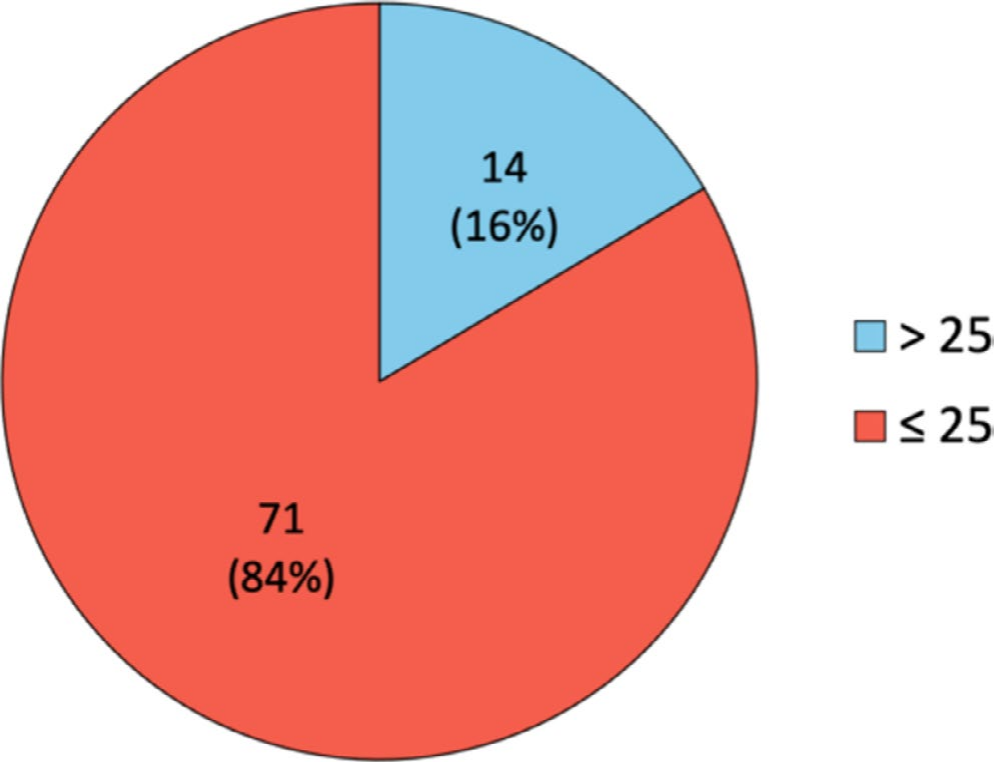
Results of the Oncotype DX tests performed (*n* = 85).

Among the 85 patients who underwent an Oncotype DX test, 14 (16%) had an RS strictly > 25, while 71 (84%) had a score ≤ 25 (Figure 2). The overall RS ranged from 3 to 56, with a median value of 18.

The tumor characteristics of patients who underwent Oncotype DX testing were collected and compared between the RS ≤ 25 and RS > 25 groups (Table 2). Among the RS ≤ 25 population, 67.6% of tumors were IDC, most were grade 2 (81.7%), and grade 3 prevalence was lower (7%) compared to RS > 25, where 71.4% of tumors were IDC, and 71.4% were grade 3. Tumors in the RS ≤ 25 group were more often pT1 (49.3% vs. 28.6%), while T2 tumors were more frequent in RS > 25 (46.5% vs. 64.3%). Lymph node involvement was higher in the RS ≤ 25 group (46.5% N1 vs. 14.3% in RS > 25). Ki67 > 20% was more frequent in RS > 25 (64.3% vs. 31%). Vascular and/or lymphatic emboli were present in 26.8% of RS ≤ 25 compared to 21.4% in RS > 25.

**TABLE 2:** Patient and disease characteristics of the RS ≤ 25 and RS > 25 subgroups.

	RS ≤ 25 population *n* = 71	RS > 25 population *n* = 14
Median of age (IQR)	74 (6)	76 (8)
IDC	48 (67.6%)	10 (71.4%)
ILC	23 (32.4%)	4 (28.6%)
pT1	35 (49.3%)	4 (28.6%)
pT2	33 (46.5%)	9 (64.3%)
pT3	3 (4.2%)	1 (7.1%)
pN0	38 (53.5%)	12 (85.7%)
pN1	33 (46.5%)	2 (14.3%)
Grade 1	8 (11.3%)	0
Grade 2	58 (81.7%)	4 (28.6%)
Grade 3	5 (7.0%)	10 (71.4%)
Ki67 > 20%	22 (31%)	9 (64.3%)
Vascular and/or lymphatic invasion	19 (26.8%)	3 (21.4%)

Abbreviations: IDC, invasive ductal carcinoma; ILC, invasive lobular carcinoma; IQR, inter‐quartile range; pN, histopathological node stage; pT, histopathological tumor stage; RS, recurrence score.

Endocrine therapy was prescribed for 5 years in patients with N0 disease and for 7 years in those with N+ disease. In the RS ≤ 25 group, seven patients (9.9%) discontinued treatment due to death or recurrence, and 11 (15.5%) due to toxicity. In the RS > 25 group, four patients (28.6%) discontinued because of death or recurrence, and two (14.9%) because of toxicity. Regarding oncogeriatric assessment in the RS > 25 population, nine patients had a consultation with a geriatric oncologist. Two patients had a G8 score > 14 and were not further evaluated. For three patients, no record of G8 evaluation or dedicated consultation was found in the medical files. Adjuvant CT was initiated in seven patients (50%). Of the seven patients eligible for CT but who did not initiate it, two were considered unfit, two refused, one experienced early relapse before initiation, and for two, the reasons were unknown. Among the seven who received CT, four were treated with the taxane cyclophosphamide regimen, and three with anthracycline cyclophosphamide—taxane regimen (AC‐T). No platinum‐based regimens were administered. Two patients discontinued CT due to poor tolerance: one due to peripheral neuropathy related to taxanes (TC regimen) and one treated with AC‐T (Table 3).

**TABLE 3:** Oncogeriatric evaluation and treatment characteristics in the RS > 25 population.

	RS > 25 population *n* = 14
Oncogeriatric assesment	
Consultation with a geriatric oncologist	9 (64.3%)
Oncodage G8 > 14 and no consultation	2 (14.3%)
None	3 (21.4%)
Adjuvant CT initiated	7 (50%)
Reason why adjuvant CT was not initiated	
Unfit	2
Refusal	2
Unknown	2
Early relapse	1
Type of CT	
AC‐T	3
TC	4
CT stopped before end of treatment	2

Abbreviations: AC‐T, anthracycline cyclophosphamide‐taxane; CT, chemotherapy; RS, recurrence score; TC, taxane cyclophosphamide (1 patient received paclitaxel and 3 patients received docetaxel).

**FIGURE 3:**
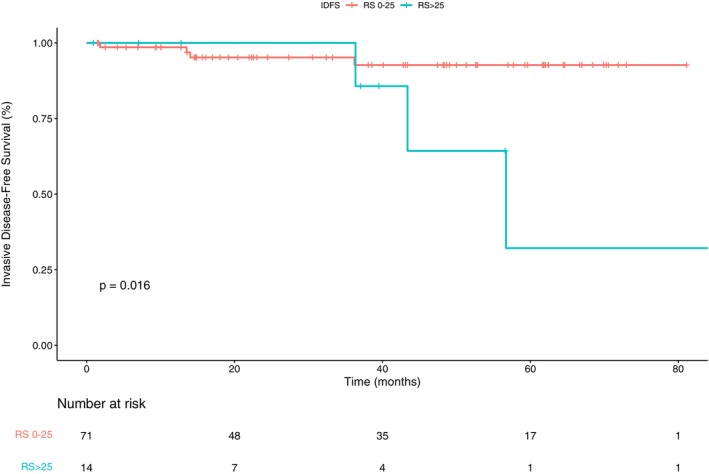
Invasive Disease‐Free Survival (IDFS) according to recurrence score (RS) in the population who underwent Oncotype DX testing (*n* = 85).

IDFS was analyzed in the Oncotype DX cohort (*n* = 85), with a median follow‐up time of 45.4 months (IQR 22.5–61.8), ranging from 0.7 to 91.4 months (Figure 3). The overall median IDFS was not reached. In subgroup analyses based on RS result, the median IDFS for patients with an RS ≤ 25 was not reached, whereas for those with an RS > 25, the median IDFS was 49.9 months (95% CI 43.4–NA). The difference in IDFS between the two groups was statistically significant (*p* = 0.016). Among the five known recurrences in the RS > 25 group, four patients did not receive adjuvant chemotherapy (two declined treatment, one was deemed unfit, and one experienced an early recurrence that did not allow time for adjuvant chemotherapy).

**FIGURE 4:**
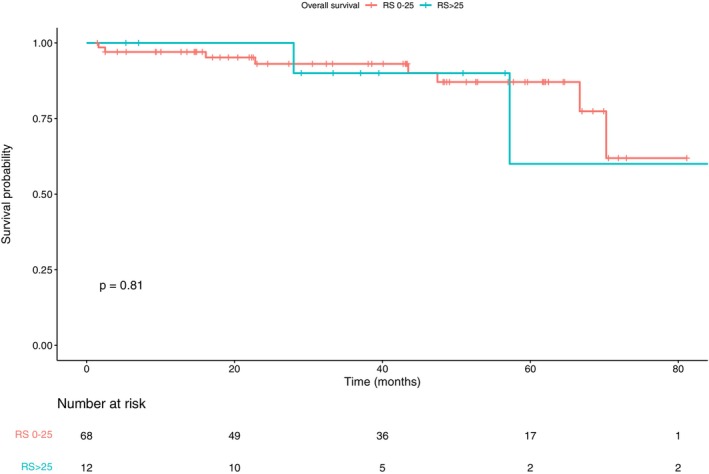
Overall survival (OS) according to recurrence score (RS) in the population who underwent Oncotype DX testing (*n* = 85).

OS analyzed 78 patients—the survival status of seven patients was unavailable—with a median follow‐up of 47.3 months (IQR 25.1–65.1) and a range of 1.5 to 91.4 months. The median OS for the Oncotype DX cohort was 87.3 months (95% CI 87.3–NR). When stratified by RS, the median OS for patients with an RS ≤ 25 was not reached, while for those with an RS > 25, the median OS was 87.3 months (95% CI 87.3–NA). The difference in OS between the two groups was not statistically significant (*p* = 0.81). Furthermore, among the 4 known deaths in the RS > 25 group, two patients died from complications related to their BC (brain metastases and carcinomatous meningitis), and one of these two patients did not receive adjuvant CT as she was deemed unfit for treatment (Figure 4).

## 4 Discussion

This prospective cohort study provides real‐world data on the use of the Oncotype DX test in women over 70 years old with HR‐positive, HER2‐negative early breast cancer (eBC), describing patient/tumor characteristics, oncogeriatric evaluations, and survival analyses (IDFS, OS) in the tested population.

Breast cancer predominantly affects older women [13]. However, pivotal trials like TAILORx and RxPONDER included only 5% and 11.6% of patients over 70, respectively [6, 7]. In TAILORx and RxPONDER menopausal subgroups, 7% and 17% of patients were over 70. Similarly, the PONDx trial—a French prospective multicenter phase 3 study evaluating the Oncotype DX BC assay in real‐life clinical practice—included only 14% of women over 70, with no subgroup analysis performed [14]. Additionally, women > 75 years were excluded from TAILORx and PONDx, limiting the generalizability of results to older populations. However, the patient characteristics in this subgroup are detailed in PONDx and align with our study: 20% had grade 3 tumors in PONDx versus 17% in ours; 19% had Ki67 > 20% in PONDx compared to 36% in our cohort.

In our study, 84% of patients who underwent Oncotype DX testing had RS ≤ 25. This aligns with the PONDx trial's > 70 years subgroup, where 52% had RS < 18 and 36% had RS results between 18 and 30. Similarly, the Israeli CLALIT database reported 80% of patients > 70 years with N0 disease had RS ≤ 25 [16]. Approximately two‐thirds of patients > 70 years were spared adjuvant chemotherapy (CT) due to the genomic assay, highlighting its role in therapeutic de‐escalation strategies.

The prognostic value of the Oncotype DX test has been consistently shown in long‐term prospective studies (TAILORx [6], WSG PlanB [17]) and in the prospective CLALIT cohort [16, 18]. An observational SEER database study confirmed the RS's prognostic value for 5‐year BC‐specific survival, even after age adjustment [19, 20]. Our investigation, through the significant results on IDFS and the trend in OS, also supports previous studies showing the prognostic value of the Oncotype DX score, with a higher recurrence rate in the RS > 25 group, even in the population over 70 years old. In the RS > 25 group, half of known deaths were due to BC progression, with one patient deemed unfit for CT later dying from BC complications. These results underscore the Recurrence Score result's prognostic value for BC recurrence and highlight the need for optimized adjuvant CT risk–benefit assessments in high‐RS patients.

Adjuvant CT's role in improving OS in older patients remains uncertain, with HR‐negative patients benefiting the most. The EBCTCG (Early Breast Cancer Trialists' Collaborative Group) meta‐analysis, encompassing 100,000 patients from 123 trials, showed reduced BC mortality with adjuvant CT, regardless of molecular status or age [21]. A US National Cancer Database retrospective cohort study of 1592 older patients with localized HR+/HER2−/N0 BC treated between 2010 and 2014 found improved OS with CT [22]. Conversely, the French ASTER 70s study, a not yet published phase III multicenter open‐label trial, comparing adjuvant CT versus no CT in similar populations, found no significant OS benefit [23]. Although our study was not designed to evaluate the impact of adjuvant CT on IDFS or OS, it is notable that 80% of recurrences (4/5) in the RS > 25 group occurred in patients who did not receive adjuvant CT, with a statistically significant association for IDFS.

Aging increases the exposure to age‐related diseases, resulting in a heterogeneous older population with large differences in fitness and frailty [24]. In our RS > 25 group, 50% did not receive adjuvant CT due to fitness concerns or treatment refusal, aligning with ASTER 70s, where only 55% of eligible and screened patients were recruited [23]. This reflects the complexity of decision‐making in this population, influenced by age, comorbidities, and patient preferences, emphasizing the importance of an optimized benefit–risk assessment of CT. In our study, CT regimens were frequently adapted for age: only one patient completed full AC‐Paclitaxel, while most received reduced regimens like taxane‐cyclophosphamide without anthracyclines.

Although dose reductions were not explicitly reported, they likely occurred at treatment initiation and were adjusted for toxicity, reflecting typical practice in older patients. Comparable data on dose reductions are not available from TAILORx, RxPONDER, or ASTER 70s, but similar challenges in CT adaptation for older patients are plausible.

The PROCURE study, a European consensus on multigene signatures in eBC management, highlighted a lack of consensus among experts regarding the use of genomic signatures like Oncotype DX [25]. It points out the complexity of interpreting and applying these tools in clinical practice. Since 2021, joint EUSOMA/SIOG guidelines state insufficient evidence supports genomic signatures in older patients [26]. Similarly, the French National Authority for Health (HAS) withdrew recommendations for Oncotype DX test use in women > 70 years as of October 2023 [27].

Our findings do not contradict these recommendations but rather illustrate the complexity of adjuvant CT decision‐making in this population, even for patients with RS > 25. Rigorous oncogeriatric assessment appears crucial in guiding decisions. For fit patients with RS > 25, adjuvant CT can be considered, but should not be systematically recommended, as its benefit remains unclear. While the Oncotype DX test serves as a clear de‐escalation tool for patients with an RS ≤ 25, representing over 80% of the cohort, its role in guiding treatment for RS > 25, despite its prognostic relevance, must be carefully weighed.

This study has several limitations. As an observational cohort, it is subject to inherent biases limiting generalizability. The Oncotype DX and RS > 25 subgroups were relatively small but comparable to the literature. Another potential bias is the ease of access to oncogeriatric evaluations in our oncogeriatric expert center, which may not be representative of smaller centers. Expert centers might be more proficient in evaluating older patients and managing chemotherapy and its complications.

This study highlights the urgent need for research on older BC patients, who remain underrepresented in pivotal trials shaping treatment guidelines. Expanding access to geriatric assessments and conducting further studies in this population are critical for improving care and informing clinical decision‐making.

## 5 Conclusion

In conclusion, in the population over 70 years old, our study demonstrates that the Oncotype DX Breast Recurrence Score test is a valuable tool for therapeutic de‐escalation in patients with RS ≤ 25 and a reliable prognostic indicator in those with RS > 25. It highlights the pivotal role of comprehensive oncogeriatric evaluation as the primary parameter for guiding adjuvant treatment strategies in older early breast cancer patients. These findings emphasize the importance of expanding access to and training in oncogeriatric assessments to optimize treatment decisions in this population. Moreover, the underrepresentation of older patients in clinical trials remains a critical gap, requiring dedicated research to refine therapeutic strategies and improve outcomes for this vulnerable demographic. The integration of geriatric oncology expertise into routine practice and the development of tailored approaches are essential to address the unique needs of older breast cancer patients.

## Author Contributions

**Clément Grosnon:** conceptualization, investigation, writing – original draft, visualization, methodology, validation, software, formal analysis, project administration, data curation. **Lauren Seknazi:** writing – review and editing, supervision, formal analysis. **Djamel Ghebriou:** writing – review and editing. **Anne Sabaila:** writing – review and editing. **David Buob:** writing – review and editing. **Mariana Nedelcu:** writing – review and editing, investigation. **Marjolaine Le Gac:** writing – review and editing. **Mathieu Jamelot:** writing – review and editing. **Coralie Prebet:** writing – review and editing. **Jean‐Pierre Lotz:** writing – review and editing, resources. **Joseph Gligorov:** conceptualization, supervision, writing – review and editing, resources. **Marc‐Antoine Benderra:** conceptualization, investigation, writing – review and editing, visualization, validation, methodology, software, formal analysis, project administration, data curation, supervision, resources.

## Ethics Statement

The study received ethical approval from the AP‐HP Data Protection Office (DPO) and was registered in the AP‐HP General Register of Data Processing on January 30, 2025 (registration number: N°20250130150713), in compliance with the General Data Protection Regulation (GDPR) requirements; it adhered to the conformity checklist for research not involving human participants. Written informed consent was obtained from all patients prior to their inclusion in this prospective study.

## Conflicts of Interest

C.G., L.S., D.G., A.S., D.B., M.N., M.L.G., M.J., C.P., J.‐P.L., and J.G. declare no conflicts of interest related to this study. M.‐A.B. has received honoraria for board membership, consulting, and symposium participation from Exact Sciences. J.G. has received honoraria for board membership, consulting, and symposium participation from Exact Sciences.

## Data Availability

The data that support the findings of this study are available from the corresponding author upon reasonable request.
